# Exocrine pancreatic dysfunction is common in hepatocyte nuclear factor 1β-associated renal disease and can be symptomatic

**DOI:** 10.1093/ckj/sfx150

**Published:** 2018-01-30

**Authors:** Rhian L Clissold, Jon Fulford, Michelle Hudson, Beverley M Shields, Timothy J McDonald, Sian Ellard, Andrew T Hattersley, Coralie Bingham

**Affiliations:** 1 Institute of Biomedical and Clinical Science, University of Exeter Medical School, Exeter, UK; 2 National Institute for Health Research Exeter Clinical Research Facility, Royal Devon and Exeter National Health Service Foundation Trust, Exeter, UK; 3 Exeter Kidney Unit, Royal Devon and Exeter National Health Service Foundation Trust, Exeter, UK

**Keywords:** developmental kidney disease, diabetes mellitus, faecal elastase, HNF1B, pancreatic hypoplasia

## Abstract

**Background:**

Heterozygous mutations in the *HNF1B* gene are the most common monogenic cause of developmental kidney disease. Extrarenal phenotypes frequently occur, including diabetes mellitus and pancreatic hypoplasia; the latter is associated with subclinical exocrine dysfunction. We measured faecal elastase-1 in patients with HNF1B-associated disease regardless of diabetes status and assessed the degree of symptoms associated with pancreatic exocrine deficiency.

**Methods:**

Faecal elastase-1 was measured in 29 patients with a known *HNF1B* mutation. We defined a low faecal elastase-1 concentration based on the 2.5 percentile of 99 healthy control individuals (410 μg/g stool). Symptoms related to pancreatic exocrine dysfunction were assessed and a subset of the *HNF1B* cohort (*n*  =  6) underwent pancreatic imaging.

**Results:**

Faecal elastase-1 was below the 2.5 percentile of the control cohort in 18/29 (62%) patients with HNF1B-associated renal disease. A total of 8/29 (28%) had a measurement suggestive of exocrine pancreatic insufficiency at  <200 μg/g stool; of these, 3 suffered with abdominal pain, loose stools and/or unintentional weight loss. All three experienced symptomatic improvement and weight gain after commencing pancreatic enzyme replacement therapy. Faecal elastase-1 was low in 7/15 (47%) HNF1B patients without diabetes compared with 11/14 (79%) of those with diabetes (P  =  0.1).

**Conclusions:**

Faecal elastase-1 deficiency is a common feature of HNF1B-associated renal disease even when diabetes is not present and pancreatic exocrine deficiency may be more symptomatic than previously suggested. Faecal elastase-1 should be measured in all patients with known HNF1B-associated disease complaining of chronic abdominal pain, loose stools or unintentional weight loss. The discovery of a low faecal elastase-1 concentration in individuals with developmental kidney disease of uncertain cause should prompt referral for *HNF1B* genetic testing.

## Introduction

Hepatocyte nuclear factor 1β (HNF1B) is a transcription factor with important roles in the development of the kidney, pancreas, liver and genital tract [1]. Heterozygous mutations of the *HNF1B* gene are the most common known monogenic cause of developmental kidney disease [2–4]. Despite this single genetic aetiology, the phenotype of HNF1B-associated renal disease is very variable (Box 1). Biochemical abnormalities, including hypomagnesaemia and hyperuricaemia, are also frequently seen [5, 6]. HNF1B-associated disease is a multisystem disorder and extrarenal phenotypic features include young-onset diabetes mellitus, pancreatic hypoplasia, abnormal liver function tests and genital tract malformations [7–12]. Genetic changes comprise either *HNF1B* intragenic mutations (one-half of patients) or an approximate 1.3-Mb deletion at chromosome 17q12, which includes the entire *HNF1B* gene [13, 14]. Both may arise spontaneously, which means there is often no family history of renal disease or diabetes [15–17]. In view of the clinical heterogeneity of the condition and frequent absence of a family history, diagnosis can be challenging and it is likely that many cases remain undetected.

**Box 1. The variable phenotype of HNF1B-associated renal disease**

- Bilateral hyperechogenic kidneys with normal or slightly increased size on antenatal ultrasonography
- Renal cysts (including cystic dysplasia and multicystic dysplastic kindney)
- Single kidney
- Renal hypoplasia
- Horseshoe kidney
- Duplex kidney
- Isolated bilateral hydronephrosis and hydroureter
- Collecting system abnormality (usually in conjunction with other renal structural abnormality)

Imaging of the pancreas in HNF1B-associated disease with either computed tomography (CT) or magnetic resonance imaging (MRI) has shown less tissue corresponding to the body and tail of the pancreas, with a slightly atrophic head [8, 9]. This is consistent with agenesis of the dorsal pancreas, the embryonic structure that gives rise to the pancreatic body, tail and a small section of the head. Pancreatic exocrine hypersecretion has been observed in patients with HNF1B-associated disease using secretin-stimulated MRI and rapid endoscopic secretin stimulation tests; this is likely to be a compensatory mechanism for reduced pancreatic volume and provides further evidence that the small pancreas seen on imaging is due to hypoplasia rather than atrophy [18]. A report of 20 foetal autopsy cases with *HNF1B* mutations described pancreatic agenesis in 2/20 and hypoplasia in 13/20 [19].

Pancreatic hypoplasia in HNF1B-associated disease has been associated with subclinical pancreatic exocrine insufficiency. This has mainly been studied in small series of patients with *HNF1B* mutations and diabetes using indirect tests of pancreatic function, usually faecal elastase-1 measurement in stool [8, 9, 20]. Lower exocrine pancreatic function involving both ductal and acinar cells has been confirmed in direct testing with rapid endoscopic secretin tests and secretin-stimulated MRI in seven individuals with *HNF1B* mutations [18]. To our knowledge, only one case of symptomatic pancreatic exocrine insufficiency requiring treatment in HNF1B-associated disease has been described in the literature to date [21]. In this study, our aims were to measure faecal elastase-1 in patients with HNF1B-associated disease regardless of diabetes status and assess the degree of symptoms associated with pancreatic exocrine deficiency.

## Materials and methods

### Recruitment and genetic analysis

Participants with HNF1B-associated disease were recruited from 31 January 2013 to 10 October 2015 from three sites in the UK (adult renal and diabetes units at the Royal Devon and Exeter Hospital; paediatric renal units at Great Ormond Street Hospital for Children and Evelina London Children’s Hospital), as previously described [22]. Inclusion criteria included the presence of either an *HNF1B* intragenic mutation or whole-gene deletion on genetic testing performed due to underlying renal abnormalities or diabetes and current age  ≥4 years. Informed written consent was obtained from all adult participants and parents of child participants, with assent from those <16 years of age. The study was conducted in agreement with the Declaration of Helsinki principles and approved by a regional ethics committee (National Research Ethics Service Committee South West—Frenchay). A total of 29 patients from 20 unrelated families with HNF1B-associated disease participated. Mutation screening was performed by sequencing of coding exons and exon–intron boundaries together with gene dosage assessment by multiplex ligation–dependent probe amplification as previously described [14, 17].

Faecal elastase-1 was also measured in a cohort of healthy controls in order to define a low faecal elastase-1 concentration based on the 2.5 percentile. Healthy controls were recruited from 4 March 2015 to 19 August 2016 from two sites in the UK (National Institute for Health Research Exeter Clinical Research Facility at the Royal Devon and Exeter Hospital; Oxford Centre for Diabetes, Endocrinology and Metabolism at the Oxford University Hospitals National Health Service Foundation Trust). Inclusion criteria included age 16–75 years, ethnicity reflective of local demographics and capacity to consent. Informed written consent was obtained from all participants. The study was conducted in agreement with the Declaration of Helsinki principles and approved by a regional ethics committee (South West—Frenchay Research Ethics Committee). A total of 99 individuals participated. The median age of this cohort was 61.7 years [interquartile range (IQR) 52.8–66.3]. A total of 39/99 (39%) was male and all were of White ethnicity. The median faecal elastase-1 concentration was 1580 μg/g stool (IQR 1000–2000). The 2.5 percentile for faecal elastase-1 in this cohort was a concentration of 410 μg/g stool (Supplementary data, Figure S1). There was only a weak association between increasing age and lower faecal elastase-1 concentrations with a Spearman’s ρ of −0.2, (P * *= * *0.02) (Supplementary data, Figure S2).

### Clinical evaluation

Relevant medical details, including symptoms related to pancreatic exocrine dysfunction (abdominal pain, loose stools and unintentional weight loss), were documented using a standardized assessment of medical records and participant/parent interview. Diabetes was diagnosed either according to World Health Organization guidelines or on the basis of established treatment with oral hypoglycaemic agents/insulin. In order to measure endogenous insulin production, the urinary C-peptide creatinine ratio (UCPCR) was measured on a post-prandial urine sample taken approximately 2 h after a meal stimulus [23].

Faecal elastase-1 concentration was assessed by enzyme-linked immunosorbent assay on a single spot stool sample at the Royal Cornwall Hospital; healthy control samples were tested using a 10× dilution to obtain an absolute value for faecal elastase-1, as the majority of samples were expected to have a result greater than the 500 μg/g detection limit of the assay. Faecal elastase-1 <200 μg/g is considered abnormal, with measurements of 100–200 μg/g suggestive of moderate to mild pancreatic exocrine insufficiency and measurements <100 μg/g suggestive of severe insufficiency [24]. Previous imaging results from CT or MRI were reviewed to look for pancreas abnormalities. All patients with HNF1B-associated disease were also invited to undergo pancreatic MRI using a 1.5-T Philips Intera system utilizing three-dimensional gradient echo and spin echo sequences, with and without fat suppression, at a range of orientations. Images were subsequently reviewed and reported by a consultant radiologist in order to assess pancreatic structure and ensure there were no incidental findings of clinical concern.

### Statistical analysis

Qualitative variables were described with percentages and quantitative variables with median and IQR. Differences between groups were assessed using the Fisher exact test for categorical variables and the Mann–Whitney *U* test for continuous variables. Correlations were tested by Spearman’s ρ. A P-value <0.05 was considered to be statistically significant. All analyses were carried out using StataSE (version 14; StataCorp, College Station, TX, USA) and GraphPad statistical software (GraphPad Software, La Jolla, CA, USA).

## Results

### Participant characteristics

The median age of individuals with HNF1B-associated renal disease was 25 years (IQR 14–44) and 13/29 (45%) were male. The majority of the cohort was White, with just 1/29 (3%) being of mixed ethnicity. A total of 14/29 (48%) had diabetes.

### Exocrine pancreatic deficiency is common in HNF1B-associated disease and can be symptomatic

**Fig. 1.**
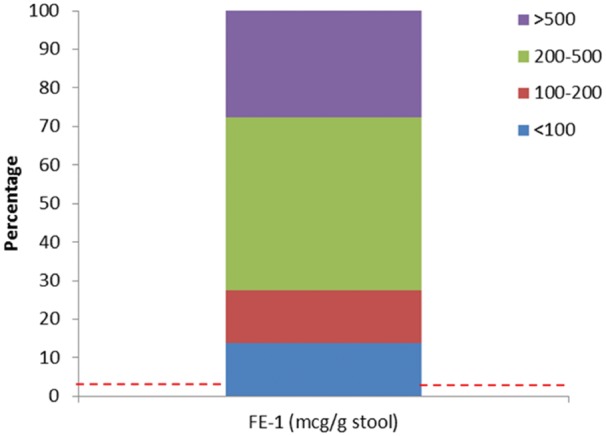
Bar chart showing the percentage of individuals with HNF1B-associated disease with faecal elastase-1 (FE-1) measurements <100 μg/g stool (suggestive of severe pancreatic exocrine insufficiency), 100–200 μg/g stool (moderate to mild insufficiency), 200–500 μg/g stool and >500 μg/g stool. The dotted red line indicates that 3/99 (3%) healthy controls had an FE-1 measurement of 200–500 μg/g stool whereas in the remainder it was >500 μg/g stool.

Faecal elastase-1 was low (below the 2.5 percentile of the control cohort) in 18/29 (62%) patients with HNF1B-associated renal disease. In all, 8/29 (28%) had a faecal elastase-1 concentration suggestive of exocrine pancreatic insufficiency at <200 μg/g stool (Figure 1); in 4/29 (14%) the measurement was <100 μg/g stool, in keeping with severe deficiency.

A total of 3/8 individuals with a faecal elastase-1 measurement <200 μg/g stool suffered with abdominal pain, loose stools and/or unintentional weight loss. All reported symptomatic improvement and weight gain after commencing pancreatic enzyme replacement therapy. In all three cases it had taken several months for symptoms to be attributed to faecal elastase deficiency and for treatment to be commenced (Table 1).

**Table 1.** Details of symptomatic faecal elastase deficiency in three individuals with HNF1B-associated renal disease

Patient	Age at study entry (years)	Sex	Genetic abnormality	Diabetes status	FE-1, μg/g stool	Pancreatic insufficiency	
						Age at diagnosis (years)	Details
2	36	Female	Mutation (c.982_986delCCTCT)	Diagnosed at age 20 years, on insulin	90	39	FE-1 measured by renal team after several months of abdominal pain and diarrhoea; resolution of symptoms and weight gain after treatment commenced
3	61	Female	Deletion (c.1-?_1674+? del)	Haemoglobin A1c of 56 mmol/mol identified at age 59 years but no treatment commenced	31	63	Known pancreatic atrophy and calcification from previous imaging for abdominal pain under surgical team. FE-1 measured by gastroenterology after referral with several months of abdominal pain, loose stools and weight loss; improvement in symptoms and 6-kg weight gain after pancreatic enzyme replacement therapy commenced
18	43	Female	Deletion (c.1-?_1674+? del)	Diagnosed at age 32 years, on insulin	107	43	Referred to gastroenterology with abdominal pain, diarrhoea (with occasional blood mixed in) and weight loss; colonoscopy performed and found to be normal. FE-1 measured by research team; commenced on pancreatic enzyme replacement therapy with symptomatic improvement

del, deletion; FE-1, faecal elastase-1.

### Individuals with low faecal elastase-1 levels have radiological evidence of pancreatic hypoplasia

In all, 6/29 participants underwent pancreatic imaging with either CT or MRI. Abnormalities were detected in four of six participants: one was reported to show diffuse pancreatic atrophy with calcification of the head and body plus common bile duct dilatation, whereas the other three demonstrated absence or atrophy of the body and tail of the pancreas only. All four patients had been diagnosed with diabetes and faecal elastase-1 measurements ranged from 31 to 280 μg/g stool. Two of six individuals had scans reported within normal limits. One of these patients was a 20-year-old male without evidence of diabetes and a normal faecal elastase-1 result of 432 μg/g stool. The other patient was a 65-year-old female who had been diagnosed with new-onset diabetes after transplantation at the age of 62 years and had a faecal elastase-1 result >500 μg/g stool.

### Markedly low faecal elastase-1 levels are more common in HNF1B-associated disease when diabetes is present

**Fig. 2.**
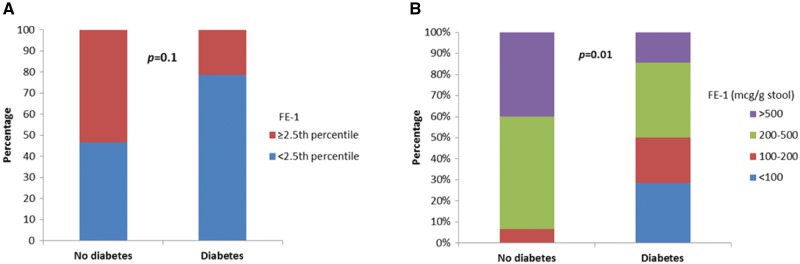
Bar charts showing the percentage of individuals with HNF1B-associated disease according to diabetes status with faecal elastase-1 (FE-1) measurements **(A)** either below or above the 2.5 percentile of a healthy control cohort and **(B)** <100 μg/g stool (suggestive of severe pancreatic exocrine insufficiency), 100–200 μg/g stool (moderate to mild insufficiency), 200–500 μg/g stool and >500 μg/g stool.

Faecal elastase-1 measurements were compared in individuals with HNF1B-associated disease according to diabetes status. Only 1/15 (7%) of those without diabetes had a markedly low faecal elastase-1 measurement (<200 μg/g stool) compared to 7/14 (50%) of those with diabetes (P * *= * *0.01) (Figure 2). Overall, faecal elastase-1 levels were low in 7/15 (47%) HNF1B patients without diabetes compared with 11/14 (79%) of those with diabetes (P * *=* * 0.1). There was only a weak association between increasing duration of diabetes and lower faecal elastase-1 concentrations, with a Spearman’s ρ of −0.3 (P * *= * *0.4) (Supplementary data, Figure S3).

The two groups were different with respect to many clinical characteristics (Table 2). The median age of the cohort without diabetes was 14 years (IQR 9–19) compared to 43.5 years (IQR 34–55) in the group with diabetes (P * *= * *0.0005). In those patients with more severe pancreatic disease, endogenous insulin secretion assessed by the UCP:Cr ratio was lower than in those without diabetes [median UCP:Cr ratio 1.1 nmol/mmol (IQR 0.6–1.5) compared to 2.1 nmol/mmol (1.4–5.6), respectively; P * *= * *0.04].

**Table 2.** Characteristics of individuals with HNF1B-associated disease according to diabetes status

Feature	HNF1B-associated disease, no DM (*n * = * *15)	HNF1B-associated disease with DM (*n * = * *14)	P-value
Age (years), median (IQR)	14 (9–19)	43.5 (34–55)	0.0005
Sex, *n* (%)	Male 9 (60), Female 6 (40)	Male 4 (29), Female 10 (71)	0.1
Genetic abnormality, *n* (%)	Mut 9 (60), del 6 (40)	Mut 7 (50), del 7 (50)	0.7
BMI (kg/m^2^), median (IQR)	20 (18–27)	23 (20–25)	0.1
Creatinine (*µ*mol/L), median (IQR)	79 (55–115)	116 (90–144)	0.03
HbA1c (mmol/mol), median (IQR)	39 (37–42)	60 (55–74)	0.0005
UCP:Cr ratio (nmol/mmol), median (IQR)	2.1 (1.4–5.6)	1.1 (0.6–1.5)	0.04

del, deletion; DM, diabetes mellitus; mut, mutation.

## Discussion

We have demonstrated that faecal elastase-1 deficiency is common in HNF1B-associated renal disease and exocrine pancreatic dysfunction may be more symptomatic than previously published. This has important implications for the screening and treatment of these patients. In all, three of eight individuals in this study with a faecal elastase-1 measurement <200 μg/g stool had abdominal pain, loose stools and weight loss. The only published report of symptomatic pancreatic insufficiency in HNF1B-associated disease involved the identification of diabetes and a small pancreas on imaging in an individual 5 years of age; pancreatic enzyme replacement therapy became necessary from the age of 16 years and lead to a normalization of body mass index [21]. There was a significant delay in attributing symptoms to pancreatic insufficiency in the three cases identified in this article, and one of the patients even underwent a colonoscopy before the correct diagnosis was made. All showed symptomatic improvement with weight gain once treatment with pancreatic enzyme replacement therapy was commenced. This highlights how difficult it can be to diagnose pancreatic insufficiency and how prompt treatment benefits patients. Clinicians should have a low threshold for arranging pancreatic function testing for individuals with known HNF1B-associated disease, even when subtle symptoms such as mild abdominal discomfort and bloating are present.

This is the largest series of faecal elastase-1 measurements in individuals with HNF1B-associated renal disease recruited irrespective of diabetes status; previous reports of faecal elastase deficiency in association with an *HNF1B* mutation have usually been from smaller series of patients with diabetes or prediabetes [8, 9, 18, 20]. Tjora *et al.* [18] included one patient with an *HNF1B* mutation and normal glucose tolerance in their study of exocrine pancreatic function using direct testing; this individual was 38 years old with normal pancreas anatomy on imaging and a faecal elastase-1 measurement of 312 μg/g stool. An earlier study from the same group recruited an affected 6-year-old girl with no pancreatic body and tail identified on imaging and a faecal elastase-1 concentration of 131 μg/g stool; she had developed impaired glucose tolerance when studied again at the age of 8 years [9, 18]. We included 15 HNF1B patients without diabetes in this study; 7/15 (47%) had a low faecal elastase-1 measurement but only 1/15 (7%) had a measurement of <200 μg/g stool. We would hypothesize that pancreatic insufficiency and diabetes in HNF1B-associated disease are associated, as they are secondary to reduced exocrine and endocrine cells as a result of pancreatic hypoplasia. However, caution must be applied when interpreting the results between the HNF1B cohorts with and without diabetes, as any differences may reflect the discrepancy in age between the two groups. It would be very interesting in future work to follow a cohort of paediatric patients with *HNF1B* mutations over time using serial indirect pancreatic function testing and imaging to see if these non-invasive tests can be used to predict who will develop diabetes and exocrine insufficiency and at what age.

Several limitations were associated with this work. The cohort of healthy controls used to define the lower limit of the normal range for faecal elastase-1 were older; the median age was 61.7 years (IQR 52.8–66.3) compared to 24.5 years (IQR 14–44) in the individuals with HNF1B-associated renal disease. However, faecal elastase concentrations decline with age, so defining a cut-off for faecal elastase-1 using the 2.5 percentile of a younger control cohort may have yielded an even greater value than the 410 μg/g stool used in this study [25]. The median faecal elastase-1 measurement of 1580 μg/g stool (IQR 1000–2000) in our local control cohort is higher than values reported for healthy controls in other studies [25–27]. This may reflect assay differences between laboratories but, as we have defined low faecal elastase-1 as measurements that fall below the 2.5 percentile of a local healthy control group, this make our results generalizable. Furthermore, we found that our local cut-off of 410 μg/g stool correlated with the presence or absence of pancreatic hypoplasia on radiological imaging. Finally, our small sample size of 29 individuals with HNF1B-associated disease means we may have been underpowered to make definitive comments on the comparison of patients with and without diabetes.

There is no consensus as to when *HNF1B* genetic testing should be performed. Two tools have been developed in recent years to help select individuals who would benefit from screening: the *HNF1B* score designed by Faguer *et al.* [28] and adapted criteria for *HNF1B* analysis proposed by Raaijmakers *et al.* [29]. However, faecal elastase was not systematically assessed in either of these studies. It is cheap and easy to measure, requiring only a single spot stool sample. Given that low faecal elastase-1 concentrations were seen in 18/29 (62%) patients with HNF1B-associated renal disease in this study, it would be interesting to test the role of faecal elastase-1 as a biomarker for HNF1B-associated disease in a large cohort of individuals with congenital anomalies of the kidneys and urinary tract. In the interim, we suggest that the finding of a low faecal elastase measurement in individuals with developmental kidney disease of uncertain cause should prompt referral for *HNF1B* genetic testing.

## Conclusion

In summary, faecal elastase-1 deficiency is an important feature of HNF1B-associated renal disease even when diabetes is not present. Faecal elastase-1 should be measured in all individuals with an *HNF1B* mutation complaining of abdominal pain, loose stools or unintentional weight loss. The role of faecal elastase-1 as a biomarker for HNF1B-associated disease requires further investigation.

## Supplementary Material

Supplementary Data
