# Supplementary material for: Exocrine pancreatic dysfunction is common in hepatocyte nuclear factor 1β-associated renal disease and can be symptomatic
Source: Clin Kidney J. 2018 Jan 30;11(4):453–8. doi: 10.1093/ckj/sfx150 (PMC6070112; doi:10.1093/ckj/sfx150)
Supplement: Supplementary Data [file sfx150_supplementary_material.docx]

**Supplementary information**


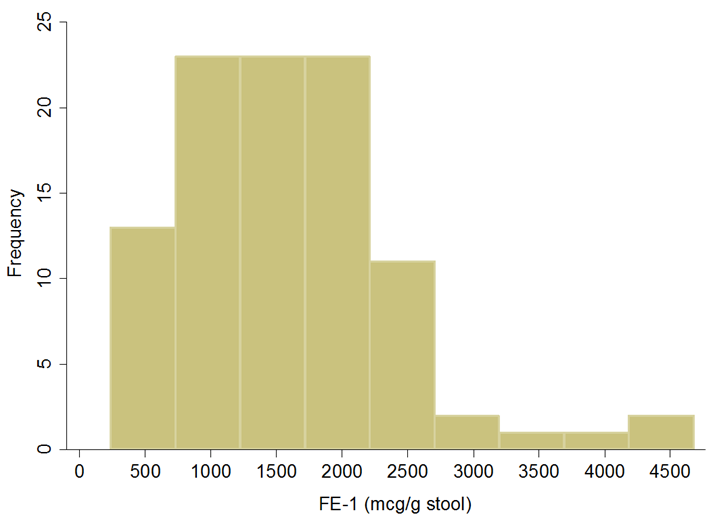


**Figure S1** Histogram of faecal elastase-1 (FE-1) concentrations in a cohort of healthy controls (*n*=99)


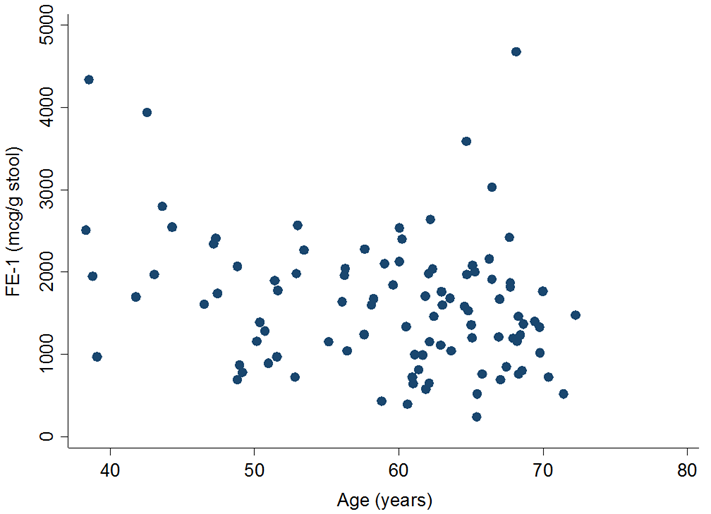


**Figure S2** Scatter plot of age versus faecal elastase-1 (FE-1) concentration in a cohort of healthy controls (*n*=99)


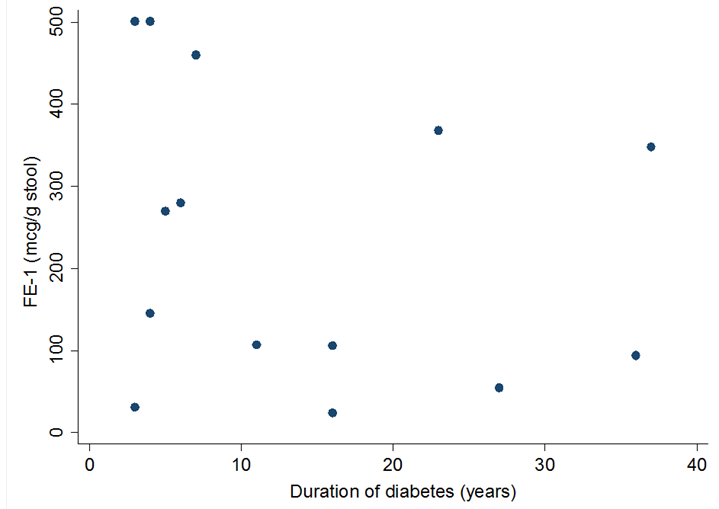


**Figure S3** Scatter plot of duration of diabetes versus faecal elastase-1 (FE-1) concentration in a cohort of patients with HNF1B-associated disease and diabetes (*n*=14); a value of 501 mcg/g stool was assigned to the two individuals with a faecal elastase-1 result of >500 mcg/g stool, although the actual value may have been higher than this
